# Straw return enhances grain yield and quality of three main crops: evidence from a meta-analysis

**DOI:** 10.3389/fpls.2024.1433220

**Published:** 2024-08-08

**Authors:** Ruipeng Zhang, Haiyang Yu, Wenbiao Zhang, Wei Li, Hao Su, Sixuan Wu, Qiong Xu, Yaying Li, Huaiying Yao

**Affiliations:** ^1^ Key Laboratory of Urban Environment and Health, Institute of Urban Environment, Chinese Academy of Sciences, Xiamen, China; ^2^ Zhejiang Key Laboratory of Urban Environmental Processes and Pollution Control, CAS Haixi Industrial Technology Innovation Center in Beilun, Ningbo, China; ^3^ University of Chinese Academy of Sciences, Beijing, China; ^4^ College of Resource and Environment, Anhui Agricultural University, Hefei, China; ^5^ Beilun District Agriculture and Rural Bureau, Ningbo, China; ^6^ Research Center for Environmental Ecology and Engineering, School of Environmental Ecology and Biological Engineering, Wuhan Institute of Technology, Wuhan, China

**Keywords:** food security, agricultural management, straw return, grain yield, grain quality, meta-analysis

## Abstract

Straw return is regarded as a widely used field management strategy for improving soil health, but its comprehensive effect on crop grain yield and quality remains elusive. Herein, a meta-analysis containing 1822 pairs of observations from 78 studies was conducted to quantify the effect of straw return on grain yield and quality of three main crops (maize, rice, and wheat). On average, compared with no straw return, straw return significantly (*p*< 0.05) increased grain yield (+4.3%), protein content (+2.5%), total amino acids concentration (+1.2%), and grain phosphorus content (+3.6%), respectively. Meanwhile, straw return significantly (*p*< 0.05) decreased rice chalky grain rate (-14.4%), overall grain hardness (-1.9%), and water absorption of maize and wheat (-0.5%), respectively. Moreover, straw return effects on grain yield and quality traits were infected by cultivated crop types, straw return amounts, straw return methods, and straw return duration. Our findings illustrated that direct straw return increased three main crop grain yields and improved various quality traits among different agricultural production areas. Although improper straw return may increase plant disease risk and affect seed germination, our results suggest that full straw return with covered or plough mode is a more suitable way to enhance grain yield and quality. Our study also highlights that compared with direct straw return, straw burning or composting before application may also be beneficial to farmland productivity and sustainability, but comparative studies in this area are still lacking.

## 1 Introduction

Food security is one of the most important challenges that humanity must face in the near future due to environmental changes and increasing population numbers (Opitz et al., 2016). Maize, rice, and wheat are three widely cultivated cereal crops globally, yielding significant quantities of straw and grain (Liu et al., 2023b). In recent years, these crops’ straw returns have been widely popularized and implemented as a farmland management policy in many areas of China (Li et al., 2018; Zhao et al., 2018). Straw return refers to the harvesting, crushing, and disposal of crop straw harvested from the field, and applying it back to the farmland (Zhang et al., 2023b). Much attention has focused on the beneficial effects of straw return, such as increasing soil organic matter and stimulating carbon (C) sequestration (Qin et al., 2021; Zhang et al., 2021b; Xing et al., 2022). Indeed, as natural manure, straw can supply various elements and organic matter to the soil, which changes the amount and composition of soil microbial communities (Qin et al., 2021; Zhang et al., 2021b; Xing et al., 2022). Meanwhile, straw return has been shown to meet the CO_2_ reduction target because the straw is mulched rather than burnt directly (Hongli et al., 2022; Lian et al., 2022; Li et al., 2023; Shuailin et al., 2023). However, crops’ straw properties are quite different, which is bound to have different effects on farmland soil properties (Ul Islam et al., 2023), and probably further lead to differences in crop grain yield and quality (Zhang et al., 2021a; Zhou et al., 2023). Unfortunately, the synthetical responses of grain yield and quality of the three major crops to straw return remain inconclusive. Furthermore, the combine influence of soil properties and straw return to grain yield and quality require further investigation.

There are a lot of traits to evaluate grain quality. For instance, the protein content and total amino acids concentration can be used as common traits to study the response of straw return on all three main crop quality (Min et al., 2011; Zhao et al., 2022). Generally, the effects of straw return on grain quality can be better evaluated through four interrelated categories: appearance quality, processing quality, cooking and eating quality, and nutritional quality (Hu et al., 2021). Currently, there have been numerous independent field experiments that investigate the impact of straw return on grain quality and yield. For instance, straw return could reduce winter wheat grain yield but increase protein content (Tan et al., 2019). However, there were also reported that although straw return increased crop grain yield, it significantly reduced grain quality, especially protein content (Gupta et al., 2022). Consequently, the independent field experimental results revealed either positive or negative effects on grain yield and grain quality due to straw return, making the overall conclusions obscure. Furthermore, some field management practices could further affect the responses of grain yield and quality to straw return, such as the type of cultivated crops, the amount of straw return, the method of straw return, etc (Zhang et al., 2021b; Zhao et al., 2022). These field management practices probably also have diverse effects on grain yield and quality by causing differences in the response of soil physical and chemical properties to straw return. Hence, the effects of straw return acted unsteadily on grain yield and various quality traits due to the multifarious states of the agricultural environment, which made the impacts of straw return on food security controversial (Ning et al., 2022; Zhang et al., 2023b). In addition, although straw return had a direct impact on soil properties, it might also have a lagged impact on grain yield and quality traits (Gai et al., 2019; Zhang et al., 2023a). Therefore, these findings indicate that the duration of straw return may have an impact on long-term outcomes.

Meta-analysis, a statistical method that synthesizes multiple independent studies on the same particular topic, has developed rapidly since it was introduced into ecology research in the 1990s (Hedges et al., 1999). It allowed for a more accurate measurement of the magnitude of effects in environmental studies and boosted the generalization of data collected from individual studies (Arnqvist and Wooster, 1995; Xia et al., 2017). Therefore, meta-analysis can be used to elucidate the responses of grain yield and quality to straw return. In this study, we hypothesize that: (1) straw return has an advantageous effect on grain yield and quality; (2) long-term straw return has a more significant impact than short-term practices; and (3) the impact of returning straw to the grain depends on multiple factors. Hence, the objective of this study is to elucidate the influence of straw return on both grain yield and quality, along with its underlying determinants, thus facilitating the advancement of sustainable food security in the future.

## 2 Materials and methods

### 2.1 Data collection and database establishment

Before March 30th, 2023, relevant research articles were searched using the Web of Science (https://apps.webofknowledge.com) and China National Knowledge Infrastructure (http://www.cnki.net) with the search terms “straw return, straw adding or straw incorporation” and “grain quality”. Peer-reviewed publications selected by the following criteria:

(1) selected published journals rather than academic degree dissertations;
(2) chose field experimental articles rather than pot experimental articles or reviews;
(3) each study comprised a control group (without straw return) and a treatment group involving straw return, focusing on grain yield and quality traits under identical environmental conditions (including the consistency of agronomic measures such as N application rate);
(4) the field experimental methods necessitate the direct return of straw to the field rather than post-composting or burning;
(5) the selected articles must provide both the mean values and the number of replications.

**Figure 1:**
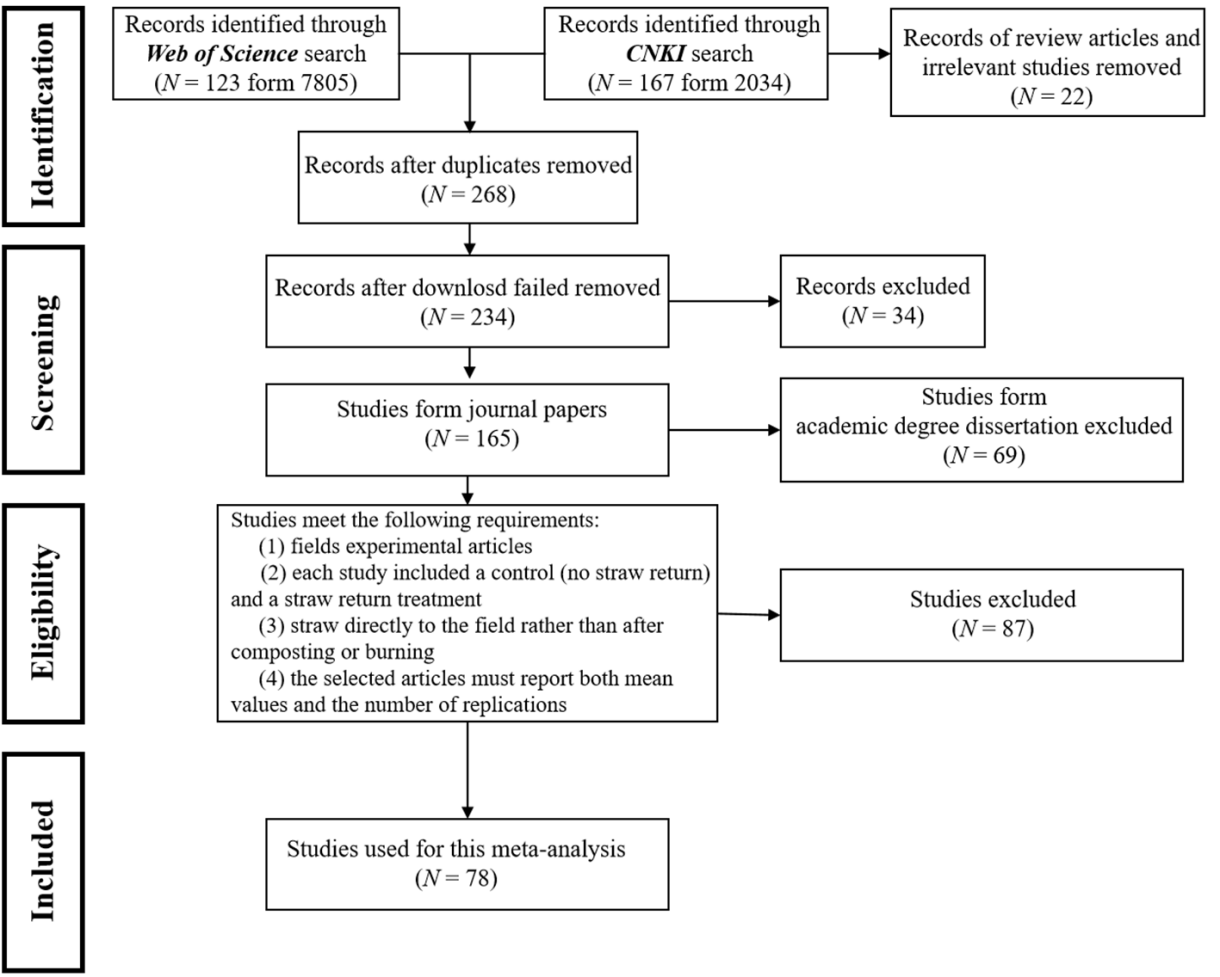
The PRISMA flow diagram of straw return effect on three main crops' grain yield and quality for this meta-analysis.

Specifically, we collected background information, grain yield data, and grain quality traits to conduct this meta-analysis. The background information included field experimental location (**Supplementary Figure S1**), type of cultivated crop, amount of straw return, method of straw return, duration of straw return, and soil properties. Most research publications reported grain yield and quality in tables that could easily be transferred into the dataset directly. For other data presented in the figures, GetData Graph Digitizer software (Version 2.24) was used to extract the data. Finally, we obtained 1822 observations from 78 articles among all preliminary screening articles (**Figure 1**; **Supplementary Text S1**).

### 2.2 Group subdivision

According to conventional classification methods, in this meta-analysis, the grain quality traits were divided into four parts: appearance quality, processing quality, cooking and eating quality, and nutritional quality. Furthermore, we used 3 traits (brown rice percentage, mill rice percentage, and flour yield) to describe grain processing quality, 3 traits (chalky grain rate, chalkiness degree, and grain volume) to describe grain appearance quality, 9 traits (amylose content, gel consistency, flavor, grain hardness, sedimentation value, resistance/extensibility relation, wet gluten content, water absorption, and oil/fat content) to describe cooking and eating quality, and 16 traits [protein content, 9 amino acids (histidine, threonine, valine, methionine, isoleucine, leucine, phenylalanine, lysine, and arginine) concentration, total amino acids concentration, and 5 grain elements (nitrogen, N; phosphorus, P; potassium, K; iron, Fe; and zinc, Zn) contents] to describe nutritional quality.

We also divided the effects of straw return on crop grain yield and quality by types of cultivated crops, amounts of straw return, methods of straw return, and durations of straw return, respectively (Yu et al., 2022). More specifically, types of cultivated crops were grouped as rice, wheat, and maize; amounts of straw return were classified into two subgroups according to the description of each search article: part straw return (≤ 3000 kg hm^-2^) and full straw return (> 3000 kg hm^-2^); methods of straw return included three modes: straw-covered mode, straw surface ploughed mode, and straw deep ploughed mode; and durations of straw return were divided into short-term (≤ 3 years), medium-term (3-10 years), and long-term (> 10 years) (Zhou et al., 2023).

### 2.3 Meta-analysis

Natural logarithms of response ratio (ln*R*) were used to measure relevant information with Equation 1: (Hedges et al., 1999; Yu et al., 2022):


(1) $$ln\;R=ln\left(M_{T}/M_{CK}\right)$$

where *M_T_* and *M_CK_* are the means of the treatment (straw return) and the control (no straw return) for each variable, respectively.

Since most of the selected studies did not report the standard deviations or standard error, we weighted the effect size as follows with Equation 2: (Xia et al., 2017; Yu et al., 2021):


(2) $$Weight=(N_{T}\times N_{CK})/\left(N_{T}+N_{CK}\right)$$

where *N_T_* and *N_CK_* are the number of replicates of the treatment and the control, respectively.

The percentage change (PC) in each variable as affected by straw return was shown using the following Equation 3: (Yu et al., 2023; Liu et al., 2023b):


(3) $$PC=\left(exp^{lnR}-1\right)\times 100\%$$

where positive *PC* means an increase whereas a negative value indicates a decrease for each variable as affected by straw return.

Fixed effect modes were conducted to generate a bootstrapping procedure with 999 iterations for mean response ratios and 95% confidence intervals (95%*CIs*) using Metawin 2.0 (Berhane et al., 2020). Between-group heterogeneity (*Q_b_* tests) was used to ensure whether the trait significantly differed among sub-groups. The Spearman test was used to ensure whether relevant factors influence the effects of straw return. The effect of long-term straw return on grain yield and quality was analyzed by linear regression. In addition, OriginPro 2024 (OriginLab, Northampton, MA, USA) was used for the Spearman test, linear regression analysis, and polynomial fit analysis to complete relevant analysis, in which a *p*-value (*, *p*< 0.05; **, *p*< 0.01; ***, *p*< 0.001) was used to judge the significance of the tests.

The Egger’s test is a statistical method used primarily in meta-analysis to assess the presence of publication bias in a set of studies. We chose Stata 16.0 (Stata Corp LLC., USA) to examines whether there is a relationship between the effect size of each study and its standard error Li et al. (2023a). The test statistic (t-value) for Egger’s test is calculated as the coefficient of the standard error term in the regression divided by its standard error, which is similar to a t-test.

## 3 Results

### 3.1 Overall effects of straw return on grain yield and quality

**Figure 2:**
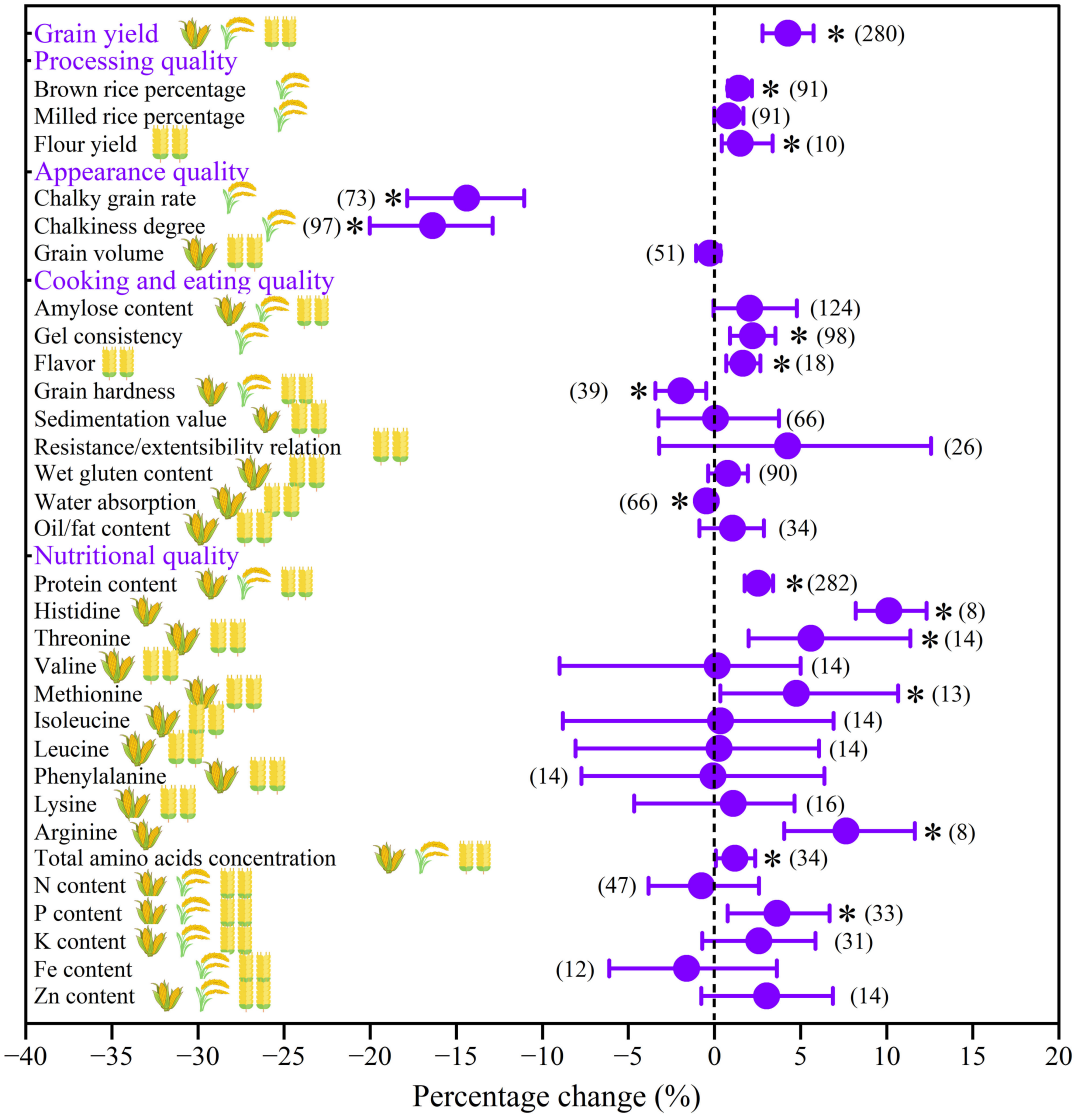
Overall effects of straw return on grain yield and quality of three main crops (maize, rice, and wheat). When the 95%CIs did not overlap with zero, the responses of variables to straw return were considered as statistically significant (p < 0.05, with asterisks). The numbers in brackets represent the number of observations.

On average, compared with no straw return, straw return increased grain yield for all three crops by 4.3% (*p*< 0.05; **Figure 2**). For grain quality, straw return improved processing quality (brown rice percentage for rice, +1.4%; flour yield for wheat, +1.5%), cooking and eating quality (gel consistency for rice, +2.2%; flavor for wheat, +1.7%), and nutritional quality (protein content, +2.5%; histidine for maize, +10.1%; threonine for maize and wheat, +5.6%; methionine for maize and wheat, +4.8%; arginine for maize, +7.6%; total amino acids concentration, +1.2%; P content, +3.6%) (*p*< 0.05, **Figure 2**). Meanwhile, straw return significantly improved rice appearance quality through decreasing chalky grain rate (-14.4%) and chalkiness degree (-16.4%) (*p*< 0.05, **Figure 2**).

### 3.2 Effects of agricultural management practices on grain yield and quality under straw return

#### 3.2.1 Types of cultivated crop affecting grain yield and quality under straw return

**Figure 3:**
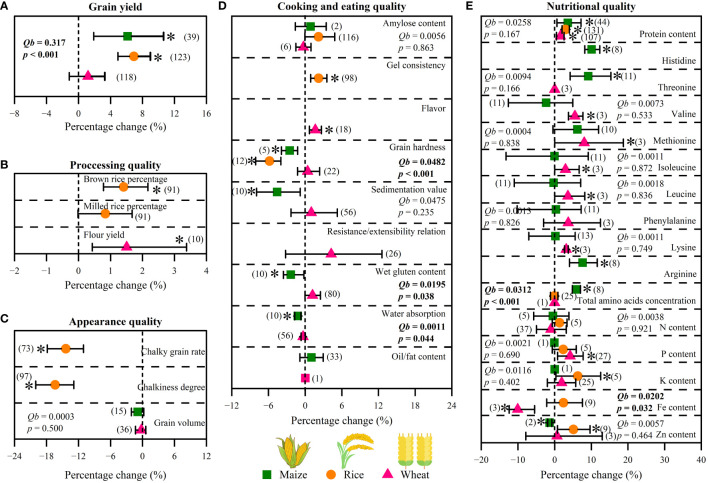
Effects of cultivated crop types (maize, rice, and wheat) on grain yield **(A)**, processing quality **(B)**, appearance quality **(C)**, cooking and eating quality **(D)**, and nutritional quality **(E)** under straw return. Between-group heterogeneity (*Qb*) represents the effects of categorical variables. Significant *Qb* values (p < 0.05) indicate that the effects of categorical variables were significant. When the 95%CIs did not overlap with zero, the responses of variables to overall fertilization were considered as statistically significant (p < 0.05, with asterisks). The numbers in brackets represent the number of observations.

The increasing effects of straw return on grain yield significantly differed among types of cultivated crop (*p*< 0.05, **Figure 3A**). Among them, straw return increased grain yield of maize and rice by 6.1% and 6.9% (*p*< 0.05), while it had no significant effect on increasing wheat grain yield (+1.2%, *p* > 0.05). For cooking and eating quality, straw return decreased grain hardness of maize and rice by 2.5% and 5.8% (*p*< 0.05), while it had no significant effect on wheat grain hardness (*p* > 0.05) (**Figure 3D**). Meanwhile, there was a significant different between maize and wheat for wet gluten content and water absorption under straw return, and their response of maize to straw return was negative (*p*< 0.05, **Figure 3D**). For nutritional quality, straw return increased total amino acids concentration of maize by 5.9% (*p*< 0.05), while it had no significant effect on that of rice and wheat (*p* > 0.05)(**Figure 3E**).

#### 3.2.2 Amounts of straw return affecting grain yield and quality

**Figure 4:**
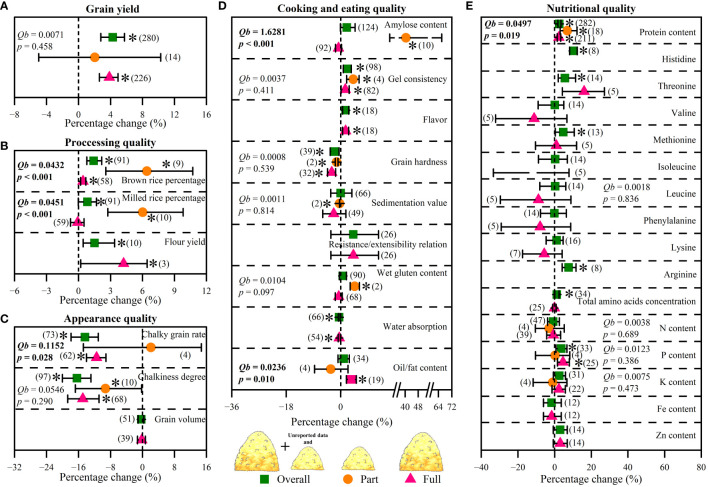
Effects of straw return amounts (part straw return and full straw return) on grain yield **(A)**, processing quality **(B)**, appearance quality **(C)**, cooking and eating quality **(D)**, and nutritional quality **(E)**. Between-group heterogeneity (*Qb*) represents the effects of categorical variables. Significant *Qb* values (p < 0.05) indicate that the effects of categorical variables were significant. When the 95%CIs did not overlap with zero, the responses of variables to overall fertilization were considered as statistically significant (p < 0.05, with asterisks). The numbers in brackets represent the number of observations.

There was no significant difference in grain yield between part and full straw return (**Figure 4A**). However, full straw return increased grain yield by 3.9% (*p*< 0.05), which was higher than that (+2.0%, *p* > 0.05) affected by part straw return (**Figure 4A**). For processing quality, the increase of brown rice percentage and milled rice percentage (+6.4% and +6.0%, respectively; *p*< 0.05) as affected by part straw return were higher than that (+0.4%, *p*< 0.05; +0.1%, *p* > 0.05) as affected by full straw return (*p*< 0.05, **Figure 4B**). For appearance quality, chalky grain rate was decreased by 11.5% (*p*< 0.05) under full straw return, while there was no significant change under part straw return (**Figure 4C**). For cooking and eating quality, amylose content was significantly increased by 40.5% (*p*< 0.05) under part straw return, while it showed insignificant change under full straw return (**Figure 4D**). Contrarily, oil/fat content significantly increased by 3.6% (*p*< 0.05) under full straw return, while there was no effect of part straw return (**Figure 4D**). For nutritional quality, protein content increased by 6.9% (p< 0.05) under part straw return, which was higher than full straw return conditions (+2.34%, p< 0.05, **Figure 4E**).

The results of linear regression analysis showed that grain yield remained unchanged as the amount of straw returned increased (*p* > 0.05, **Supplementary Figure S2A**). However, brown rice percentage, milled rice percentage, and amylose content decreased with increasing amounts of straw return (*p*< 0.001, **Supplementary Figures S2B, C, H**), while the content of isoleucine and total amino acid concentration increased with increasing amounts of straw return (*p*< 0.01, **Supplementary Figures S2V, AA**).

#### 3.2.3 Methods of straw return affecting grain yield and quality

**Figure 5:**
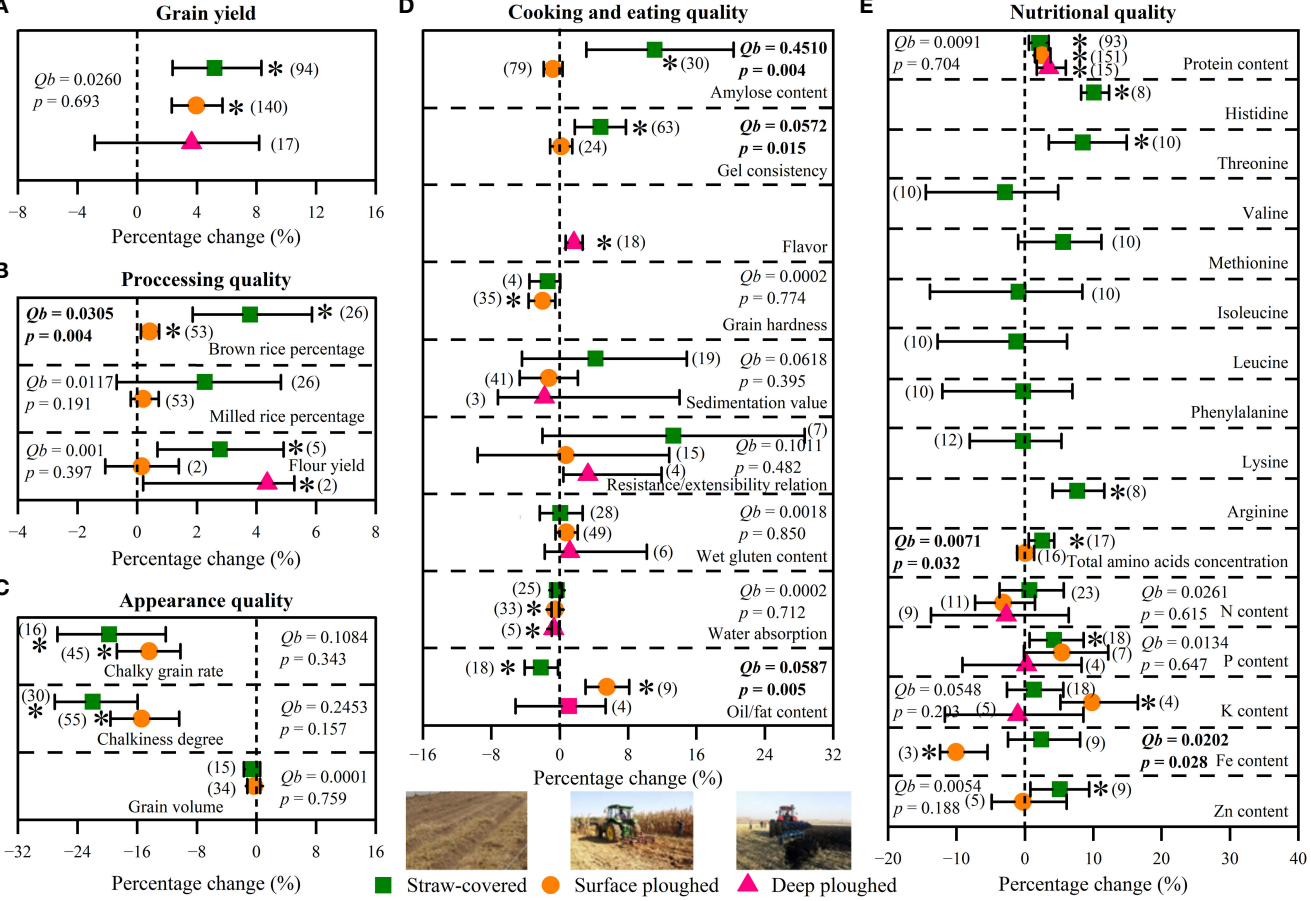
Effects of straw return methods (straw-covered mode, straw surface ploughed mode, and straw deep ploughed mode) on grain yield **(A)**, processing quality **(B)**, appearance quality **(C)**, cooking and eating quality **(D)**, and nutritional quality **(E)**. Between-group heterogeneity (*Qb*) represents the effects of categorical variables. Significant *Qb* values (p < 0.05) indicate that the effects of categorical variables were significant. When the 95%CIs did not overlap with zero, the responses of variables to overall fertilization were considered as statistically significant (p < 0.05, with asterisks). The numbers in brackets represent the number of observations.

On average, different methods of straw return had no difference in the increase in grain yield (*p* > 0.05, **Figure 5A**). However, straw-covered mode and straw surface ploughed mode increased grain yield by 5.2% and 4.0%, respectively (*p*< 0.05, **Figure 5A**), while straw deep ploughed mode did not significantly increase grain yield (+3.6%, *p* > 0.05). For processing quality, brown rice percentage significantly increased by 3.8% (*p*< 0.05) under straw-covered mode, which was higher than that under straw surface ploughed mode (+0.4%, p< 0.05, **Figure 5B**). For cooking and eating quality, amylose content and gel consistency increased by 11.1% and 4.8% under straw-covered mode, respectively (*p*< 0.05, **Figure 5D**), which was higher than that under straw surface ploughed mode, respectively (*p*< 0.05). Oil/fat content decreased under straw-covered mode by 2.2%, while it increased under straw surface ploughed mode by 5.5% (*p*< 0.05, **Figure 5D**). For nutritional quality, total amino acids concentration only increased under straw-covered mode by 2.5% (*p*< 0.05), while it showed insignificant change under straw surface plough mode (**Figure 5E**).

#### 3.2.4 Duration of straw return affecting grain yield and quality

**Figure 6:**
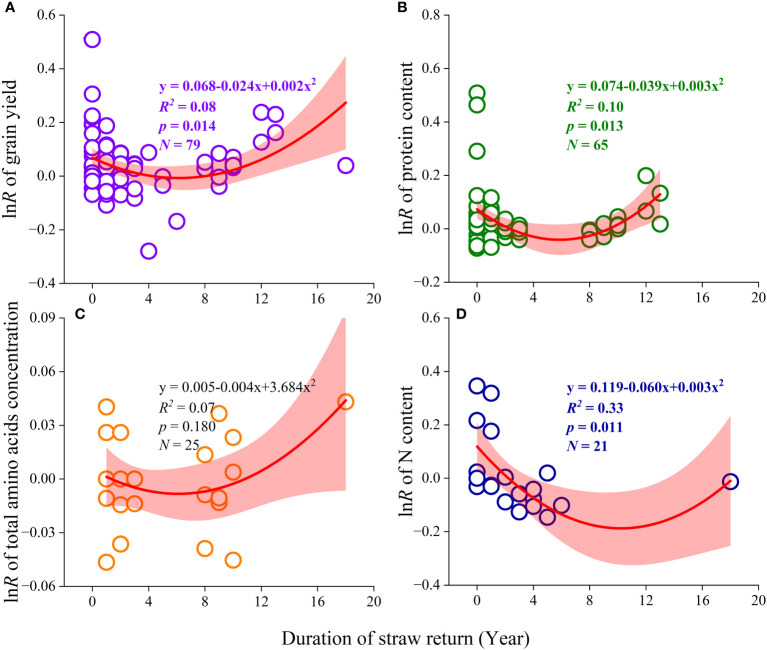
Parabolic relationship of the response ratio (lnR) of grain yield and quality with the duration of straw return. **(A)** grain yield; **(B)** protein content; **(C)** total amino acids concentration; **(D)** nitrogen (N) content. The points represent the observations and the shaded areas around the regression lines represent the 95% confidence intervals (the 95*CIs*).

There was a significant difference in grain yield among different durations of straw return (**Supplementary Figure S3**). Short- and long-term straw return increased grain yield by 6.2% and 17.3% (*p*< 0.05), respectively, while medium-tern straw return did not increase grain yield (-0.2%, *p* > 0.05) (**Supplementary Figure S3A**). Similar to grain yield, the stimulated effects of long-term straw return (+11.0%, *p*< 0.05) on protein content were higher than that (+4.8%, *p*< 0.05; -0.1%, *p* > 0.05) affected by short- and medium-term straw return, respectively (*p* > 0.05 and *p*< 0.05, **Supplementary Figure S3E**). In this study, we selected the four most important variances (grain yield, protein content, total amino acids concentration, and N content) to characterize the effects of straw return durations on grain yield and quality (**Figure 6**). In general, the promotion effects of straw return on these four most important factors first decreased and then increased with increasing the duration of straw return.

### 3.3 Relationships between fertilizer application and environmental factors with grain yield and quality as affected by straw return

**Figure 7:**
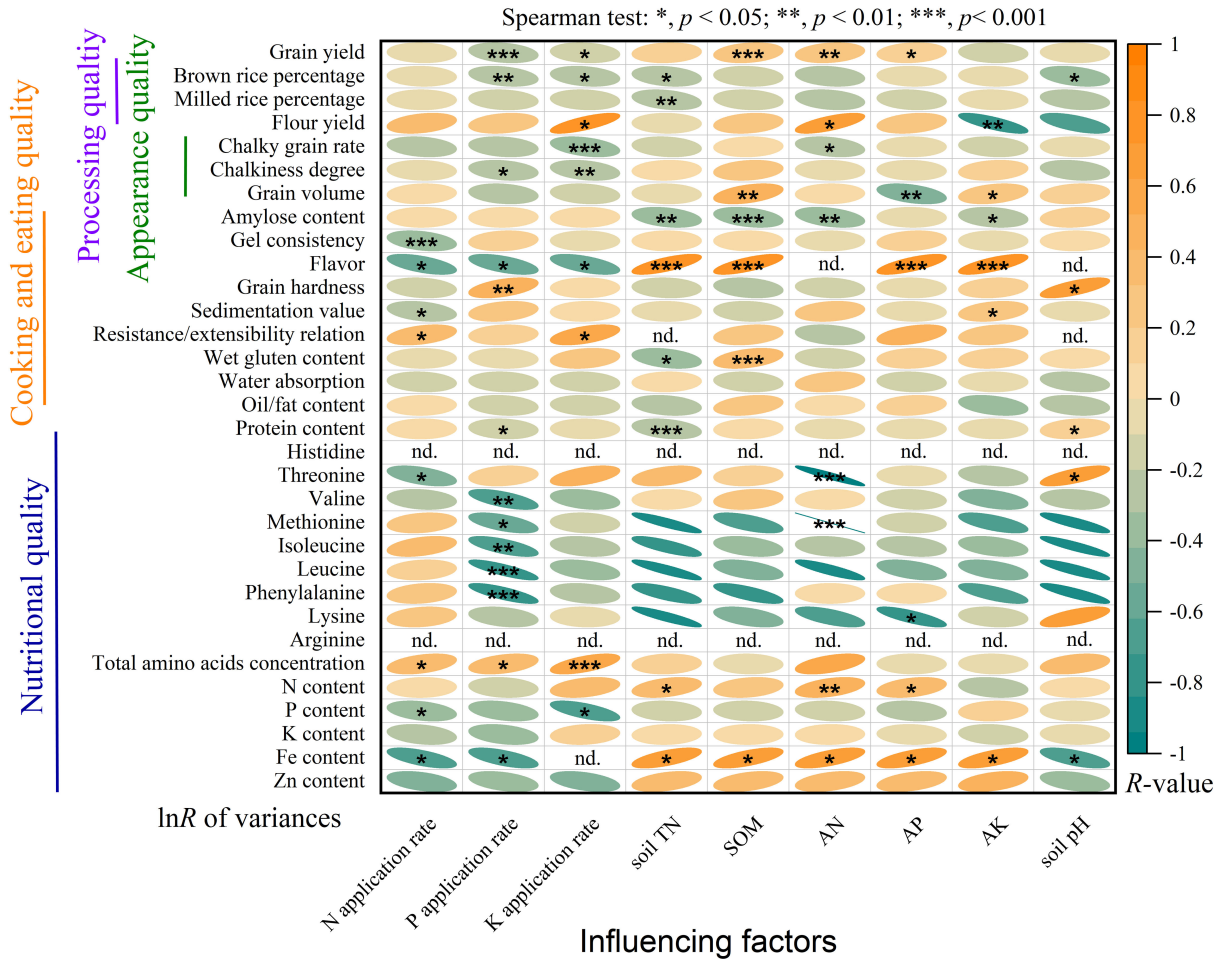
Correlation between the effect size of variances and environmental factors with Spearman test. A positive (negative) R-value denotes a positive (negative) relationship (*, p < 0.05; **, p < 0.01; ***, p < 0.001). "n.d" means "not detected".

Generally, chemical fertilization application weakened the effect of straw return on grain yield and some quality traits but increased the total amino acids concentration as affected by straw return (**Figure 7**). Furthermore, there is a positive correlation between soil fertility index with grain yield, N content, and Fe content under straw return conditions (**Figure 7**).

### 3.4 The Egger’s test conclusion for publication bias

The Egger’s test results showed (**Table 1**) that the publication bias in the study of chalkiness degree, gel consistency, flavor, histidine, threonine, arginine. Type I error for the Egger’s test was higher than other tests, which might reject the true results due to publication bias Li et al. (2023a).

**Table 1:** The Egger’s test conclusion for publication bias.

	The P-value of Egger’s test	The original conclusion	The conclusion without publication bias
Yield	0.1	*	*
Brown rice percentage	0.02	*	
Milled rice percentage	0.009		
Flour yield	0.088	*	*
Chalky grain rate	0.249	*	*
Chalkiness degree	0.001	*	
Grain volume	0.201		
Amylose content	0.235		
Gel consistency	0	*	
Flavor	0.004	*	
Grain hardness	0.719	*	*
Sedimentation value	0.012		
Resistance/extensibility relation	0.442		
Wet gluten content	0.161		
Water absorption	0.08	*	*
Oil/fat content	0.004		
Protein content	0.777	*	*
Histidine	0	*	
Threonine	0.033	*	
Valine	0.428		
Methionine	0.107	*	*
Isoleucine	0.945		
Leucine	0.951		
Phenylalanine	0.496		
Lysine	0.605		
Arginine	0.007	*	
Total amino acids concentration	0.056	*	*
N	0.001		
P	0.158	*	*
K	0.158		
Fe	0.011		
Zn	0.478		

We used the Egger’s test (H0: no small-study effects.) to test the publication bias in our result, p > 0.05 means without publication bias. “*”means the effect of straw return was significant (p > 0.05).

## 4 Discussion

### 4.1 Response of grain yield to straw return

Consistent with our hypothesis (1), straw return has a positive effect on grain yield, with a significant increase of 4.3% (95%*CIs*: 2.8%-5.8%, *p*< 0.05, **Figure 2**). Indeed, straw return not only releases more soil C, N, and P but also improves soil properties and reduces soil bulk density, which has a promoting effect on crop growth and thus increases grain yield (Lu et al., 2009; Lou et al., 2023). In addition, straw return further increases soil organic C contents by increasing microbial activity, which plays an important role in promoting soil health and high-quality crop growth (Zhou et al., 2023). Compared with previous meta-analysis studies (**Supplementary Table S1**), although the overall effect of grain yield response to straw return in this study was a reasonable magnitude, there are also some differences in grain yield among cultivated crop types (**Figure 3A**), which was consistent with previous research results (Liu et al., 2019, 2023a) (**Supplementary Table S1**). Two reasons may have caused this difference: (1) compared with maize and rice, wheat undergoes an overwintering period, while lower temperatures and precipitation are not conducive to straw decomposition and mineralization, providing fewer nutrients for wheat under straw return; (2) crops have different uptake preferences for various N forms, while (Chen et al., 2018; Quan et al., 2021) maize and rice are nitrate- and ammonium-loving crops respectively, which results in the nutrient form released by the straw after returning to paddy fields and uplands can meet the needs of their respective crops.

Crop straw, as an organic fertilizer, can change soil C/N ratio, and provide essential elements and organic C to soil (Pal and Mahajan, 2017; Zhou et al., 2020). Hence, straw return can restore the soil environment of excessive fertilization and increase grain yield concurrently (Zhang et al., 2017; Xu et al., 2022; Chen et al., 2023). Our meta-analysis showed that compared with part straw return, full straw return increased grain yield significantly (**Figure 4A**), which is probably due to being more conducive to improving soil fertility and the growth of crop roots under full straw return. Moreover, the straw-covered mode and straw surface ploughed mode were more beneficial in increasing grain yield than the deep ploughed mode of straw return (**Figure 5A**). This might be because the appropriate straw return method could accelerate the decomposition rate of straw, increase soil organic carbon content, and improve the activity of soil microorganisms (Lu et al., 2009; Zhao et al., 2016; Chen et al., 2017). Li et al. (2013) reported the accumulation of toxic substances and pathogens caused by a slow straw decomposition rate with the deep ploughed mode of straw return, which affects the effects of straw return and seed germination. Therefore, it is necessary to emphasize a proper way of straw return to improve soil environment conditions and boost grain yield. Interestingly, the effect of straw return on grain yield showed an upward parabolic relationship with straw return duration (**Figure 6A**). This indicated that long-term straw return might have more favorable effects on crop grain yield (**Supplementary Figure S3A**), which was consistent with our hypothesis (2). In summary, this finding suggests that under the condition of full straw return, long-term straw-covered mode and straw surface ploughed mode may have a better grain yield increase.

### 4.2 Response of grain quality to straw return

Similar to the response of grain yields, overall, the four types of grain quality traits also had a positive response to straw return (**Figure 2**), which was consistent with our hypothesis (1). For processing quality, the remarkable increase in brown rice percentage by straw return indicated that grains were prone to improve weight during the processing period (**Figure 2**), which produced more grain yield along the kernel surface rich in lipid and thus positively affected the commercial value (Coradi et al., 2021). Flour yield for wheat also showed the same tendency as that of brown rice percentage (**Figure 2**), indicating that straw return had a significant effect on the overall processing quality of wheat. For appearance quality, according to relevant standards, the high value of chalky grain rate and chalkiness degree represent the poor quality of rice grain (Cheng et al., 2003; Aoki et al., 2017). However, in the present study, straw return decreased the chalky grain rate and chalkiness degree (**Figure 2**), which indicated that straw return increased the appearance quality of rice (Zeng et al., 2013; Chen et al., 2014). For cooking and eating quality, in general, the greater the gel consistency of rice, the softer the rice, and the better cooking and eating quality (Cagampang et al., 1973). In this study, straw return increased the gel consistency of rice grain (**Figure 2**), resulting in a tendency to improve the taste quality of rice when cooked (Hou et al., 2015). More importantly, straw return also played a positive role in improving the nutritional quality of three main crop grains, including significantly increasing protein content, threonine, methionine, total amino acids concentration, and P content (**Figure 2**). Previous studies found that straw return promoted the transport of N to grain in the middle and late stages of rice production (Thuy et al., 2008; Wang et al., 2018; Sharma et al., 2021), which might be one of the reasons for the increase of protein content as affected by straw return.

N, P, and K are the most commonly applied chemical element in fertilizer and their effects on nutrient distribution in cereal grains have also been widely studied (Gai et al., 2019). In this study, these fertilization rates did not mediate the effects of straw return on grain N, P, and K content (**Figure 7**). This might be because straw return did not regulate the transport and conversion of crop part nutrient elements. However, there was a significant positive correlation between total amino acid concentration and fertilizer application rates (**Figure 7**), which was consistent with our hypothesis (3). Moreover, some grain quality traits were significantly correlated with others as affected by straw return (**Supplementary Figure S4**), indicating associated benefits for various quality traits by implementing straw return management (Yadvinder et al., 2004; Yang et al., 2016). For types of cultivated crops, straw return could improve various grain traits of all three crops, especially protein content and total amino acid concentration (**Figure 3**). Among them, straw return has the most significant effect on improving maize grain quality, which may be due to the highest decay rate of straw when planting maize resulting in the rapid transport of nutrient elements. For straw return types and methods, similar to the effect in grain yield, part straw return with straw-covered mode and straw surface ploughed mode was a more suitable straw return management to improve crop grain quality (**Figures 4**, **5**). Interestingly, similar to grain yield, the effect of straw return on protein content, total amino acids concentration, and N content also showed an upward parabolic relationship with straw return duration (**Figure 6A**). In other words, long-term straw return might have more favorable effects on crop grain yield (**Supplementary Figure S3A**), which was consistent with our hypothesis (2).

We utilizing the Egger’s test to evaluate publication bias within this meta-analysis, our findings demonstrate that only analyses pertaining to flour yield, chalky grain rate, grain hardness, water absorption, protein content, methionine, total amino acids concentration, and P without publication bias. It is important to acknowledge that the Egger’s test introduces the potential for Type I error Li et al. (2023a). Additionally, it is conceivable that other traits could be affected by the implementation of straw return practices, though further inclusion of study samples is necessary to confirm this hypothesis. Our study corroborates that straw return practices do indeed influence flour yield, chalky grain rate, grain hardness, water absorption, protein content, methionine, total amino acids concentration, and P on grain quality.

### 4.3 Limitations of this study

In this meta-analysis, we quantified the effects of straw return on three main crops’ grain yield and quality traits and validated that straw return enhanced overall grain yield and quality (**Figure 2**). However, there were still some limitations in our study. First, most of the previous studies we selected were conducted under short-term conditions, which made the depiction of the long-term effects of straw return on grain yield and quality hard (**Supplementary Figure S3**). Our results showed that crop grain yield and quality might first decrease and then increase with increasing the duration of straw return (**Figure 7**). This finding indicates that future studies need to conduct in-depth monitoring of the long-term effects of straw return to effectively evaluate the response of grain yield and quality to straw return (Li et al., 2023b). Unfortunately, it is undeniable that straw return would bring an increase in the risk of crop diseases and pests in the second cultivation year, thus leading to an increase in the input of pesticides for crop cultivation and protection (Liu et al., 2019). Second, straw return also could lead to changes in soil C/N ratio (Kong et al., 2023) and a further increase in N fertilizer application to ensure the efficient utilization of nutrients (Bossolani et al., 2023). This is because in low C/N ratio soils, straw return can enhance soil C sources, stimulate microbial activity, and facilitate C-driven N processes, while in high C/N ratio soils, straw decomposition by soil microbes may deplete N elements, necessitating increased N fertilizer application to ensure optimal crop growth. Thus, it is necessary to study the synergistic effect of long-term fertilization management measures and straw return. Third, for grain yield, although there are many reports on the impact of straw return on crop yield, it should be noted that there are still few studies on the comprehensive impact of straw return on food crop production, including its overall economic and ecological effects, such as input costs, greenhouse gas emissions, soil fertility, disease and pest control, and crop grain quality (Lv et al., 2019). In traditional agriculture, straw is burned in the field, and then completely mineralized, which also leaves nutrients and C in the soil, and straw incineration also effectively kills farmland pests and soil germs (Lv et al., 2019). The nutrients after the complete mineralization of straw incineration are more easily utilized by crops. Although it will increase CO_2_ emissions in the short term, in the long run, on the whole, straw incineration will not increase additional CO_2_ emissions, and the total amount should still be balanced (Kong et al., 2023). Hence, the differential effect and mechanism between straw incineration or composting before application and direct straw return should be further clarified. Meanwhile, to respond to national policies, previous studies mostly focused on the comparison of effects between reduced N fertilizer rate and no N fertilizer (or normal N fertilizer rate) treatment, and there was a lack of comparison of effects of straw return under the same N fertilizer application rate. Therefore, comparative field research in this scope should be strengthened. In addition, in terms of grain quality, due to the lack of selected data, we only collected crucial quality traits from all of the quality traits to analyze (**Figures 3**–**6**). Therefore, it is urgent to supplement experimental data to improve the impact of straw return on grain quality in the future.

## 5 Conclusion

This meta-analysis presented statistical evidence that direct straw return enhanced grain yield and quality. Specifically, straw return significantly (*p*< 0.05) increased overall grain yield (+4.3%), flour yield (+1.5%), protein content (+2.5%), methionine (+4.8%), arginine (+7.6%), total amino acids concentration (+1.2%), and the phosphorus of grain (+3.6%), compared with no straw return treatment, respectively. In addition, direct straw return significantly decreased the chalky grain rate, grain hardness, and water absorption by 14.4%, 1.9%, and 0.5%, respectively. Through subgroup analysis, we found that the effects of straw return on grain yield and quality traits were influenced by cultivated crop type, the amount of straw return, and the method of straw return. Interestingly, there was an upward parabolic relationship between grain yield and quality with straw return duration. Our study indicated that although improper direct straw return may increase plant disease risk and affect seed germination, full straw return with covered or plough mode is a more suitable way to enhance grain yield and quality under long-term straw return duration. Nevertheless, the comparative effects of direct straw return versus straw burning or composting before application on crop yield and quality deserve further study.

## Data availability statement

Our dataset has been archived and is permanently stored on KuaiPan. The data can be accessed via the following link: ‘https://pan.quark.cn/s/b80d30804e93’.

## Author contributions

RZ: Data curation, Investigation, Methodology, Software, Writing – original draft. HaY: Conceptualization, Funding acquisition, Writing – review & editing. WZ: Investigation, Writing – review & editing. WL: Data curation, Visualization, Writing – review & editing. HS: Validation, Visualization, Writing – review & editing. SW: Data curation, Writing – review & editing. QX: Data curation, Software, Writing – review & editing. YL: Funding acquisition, Supervision, Writing – review & editing. HuY: Funding acquisition, Writing – review & editing.
