# Supplementary material for: Straw return enhances grain yield and quality of three main crops: evidence from a meta-analysis
Source: Front Plant Sci. 2024 Aug 8;15:1433220. doi: 10.3389/fpls.2024.1433220 (PMC11340517; doi:10.3389/fpls.2024.1433220)
Supplement: Supplementary file 1 [file DataSheet_1.docx]

**Supplementary Materials for**

# Straw returning enhances grain yield and quality of three main crops: Evidence from a meta-analysis

Ruipeng Zhang ^a, b, c^, Haiyang Yu ^d, *^, Wei Li ^e^, Hao Su ^a, b, f^, Sixuan Wu ^a, b, c^, Qiong Xu ^e^, Yaying Li ^a, b, **^, Huaiying Yao ^e^

*^a^ Key Laboratory of Urban Environment and Health, Ningbo Observation and Research Station, Institute of Urban Environment, Chinese Academy of Sciences, Xiamen 361021, China*

*^b^* *Zhejiang Key Laboratory of Urban Environmental Processes and Pollution Control, CAS Haixi Industrial Technology Innovation Center in Beilun, Ningbo 315830, China*

*^c^ University of Chinese Academy of Sciences, Beijing 100049, China*

*^d^ College of Resource and Environment, Anhui Agricultural University, Heifei 230036, China*

*^e^ Research Center for Environmental Ecology and Engineering, School of Environmental Ecology and Biological Engineering, Wuhan Institute of Technology, Wuhan 430073, China*

*^f^ College of Resources and Environment, Fujian Agriculture and Forestry University, Fuzhou 350002, China*

^*^ Corresponding author: H. Yu, College of Resource and Environment, Anhui Agricultural University, Heifei 230036, China.

^**^ Correspondence author: Y. Li, Key Laboratory of Urban Environment and Health, Ningbo Observation and Research Station, Institute of Urban Environment, Chinese Academy of Sciences, Xiamen 361021, China

E-mail address: hyyu@ahau.edu.cn (H. Yu); yyli@iue.ac.cn (Y. Li).

**Contents:**

Number of pages: 17

Supplementary table: Table S1

Supplementary figures: Fig. S1-S4

Supplementary Test: Text S1


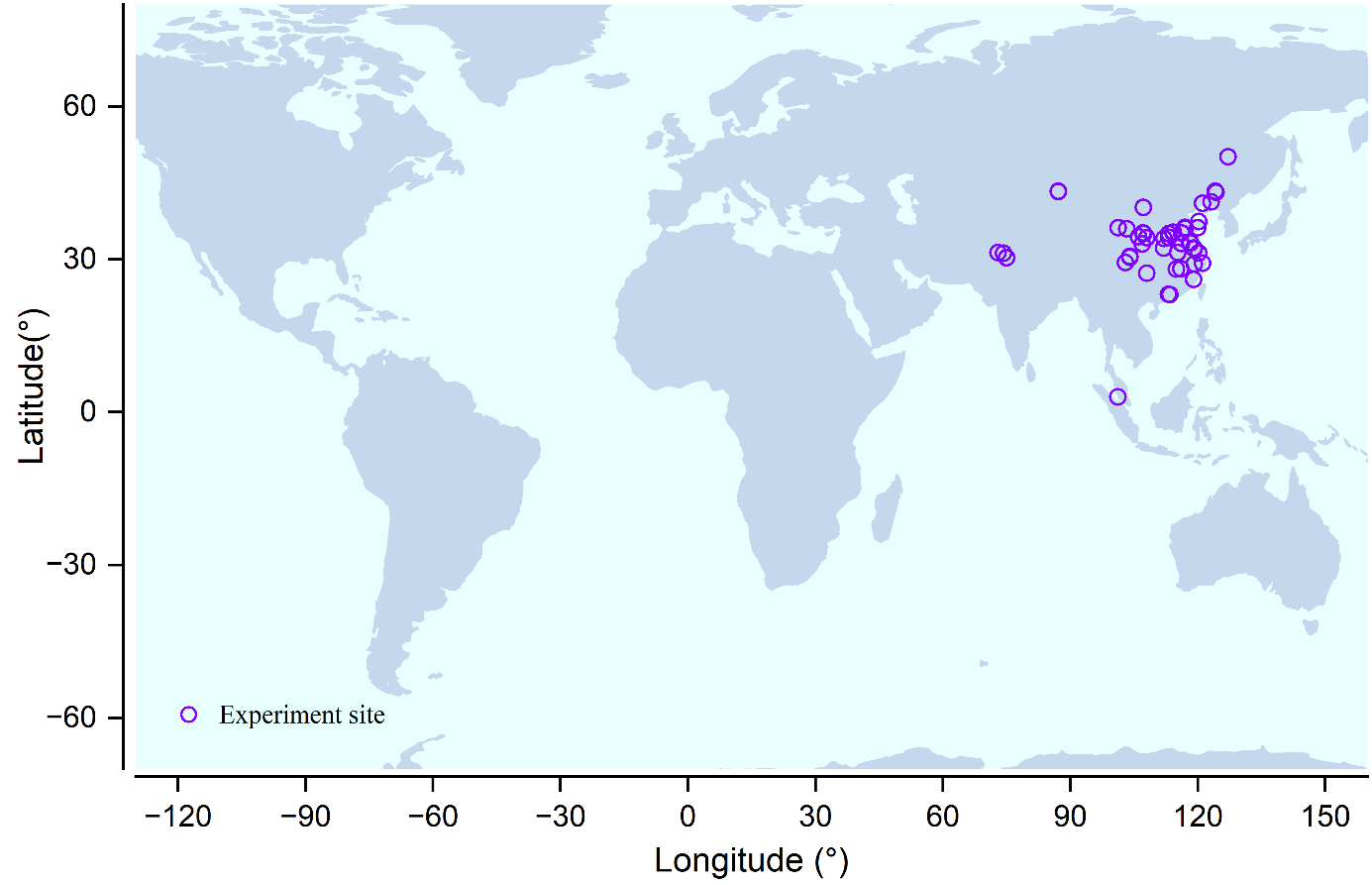


**Fig. S1** Geographical locations of the experimental sites in this meta-analysis.


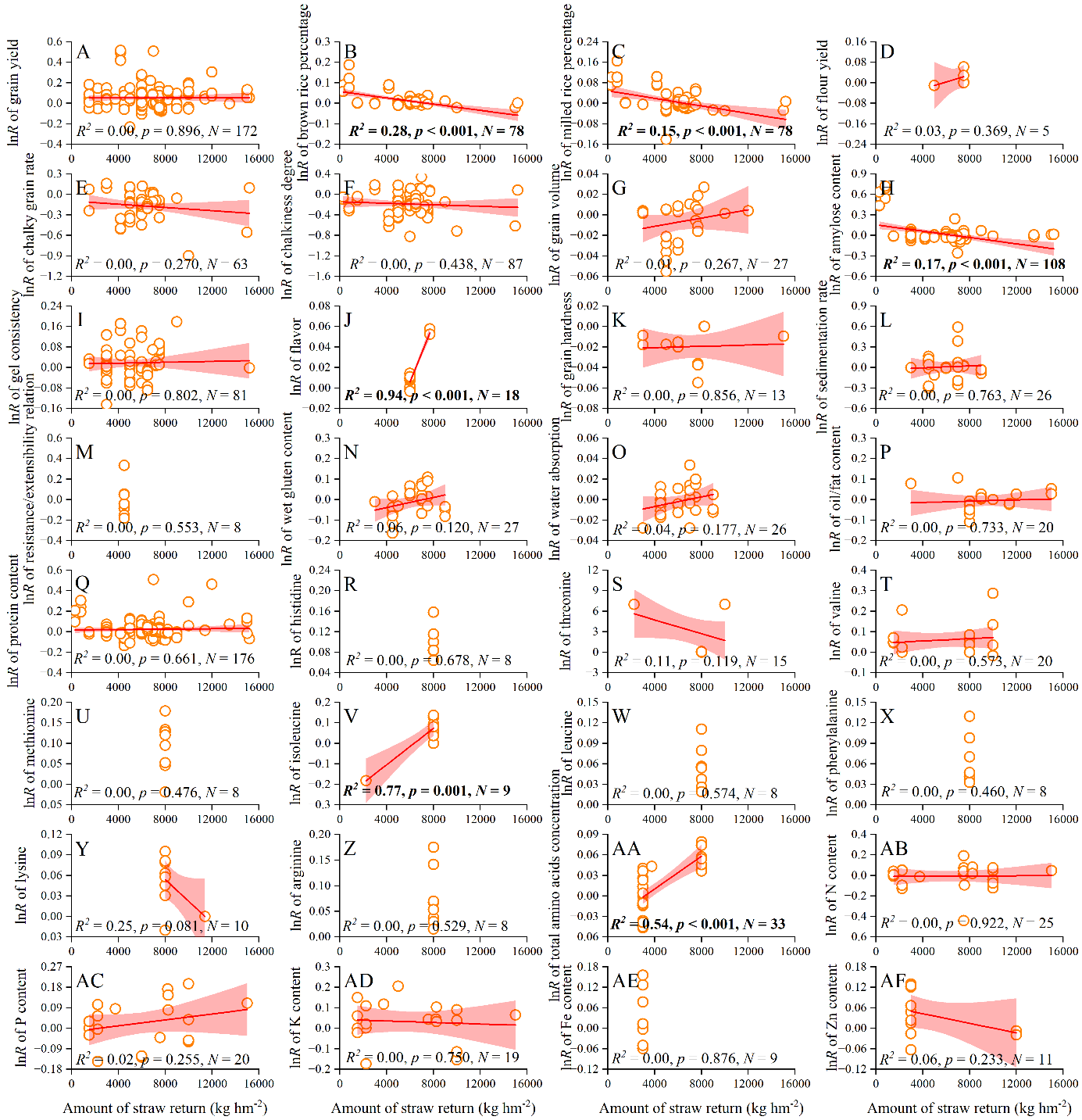


**Fig. S2** Relationship between the response ratio (ln*R*) of grain yield and quality traits with the amount of straw return. A, grain yield; B, brown rice percentage; C, milled rice percentage; D, flour yield; E, chalky grain rate; F, chalkiness degree; G, grain volume; H, amylose content; I, gel consistency; J, flavor; K, grain hardness; L, sedimentation value; M, resistance/extensibility relation; N, wet gluten content; O, water absorption; P: oil/fat content; Q, protein content; R, histidine; S, threonine; T, valine; U, methionine; V, isoleucine; W, leucine; X, phenylalanine; Y, lysine; Z, arginine; AA, total amino acids concentration; AB, nitrogen (N) content; AC, phosphorus (P) content; AD, potassium (K) content; AE, Iron (Fe) content; AF, Zinc (Zn) content. The points represent the observations and the shaded areas around the regression lines represent the 95% confidence intervals (the 95%*CIs*).


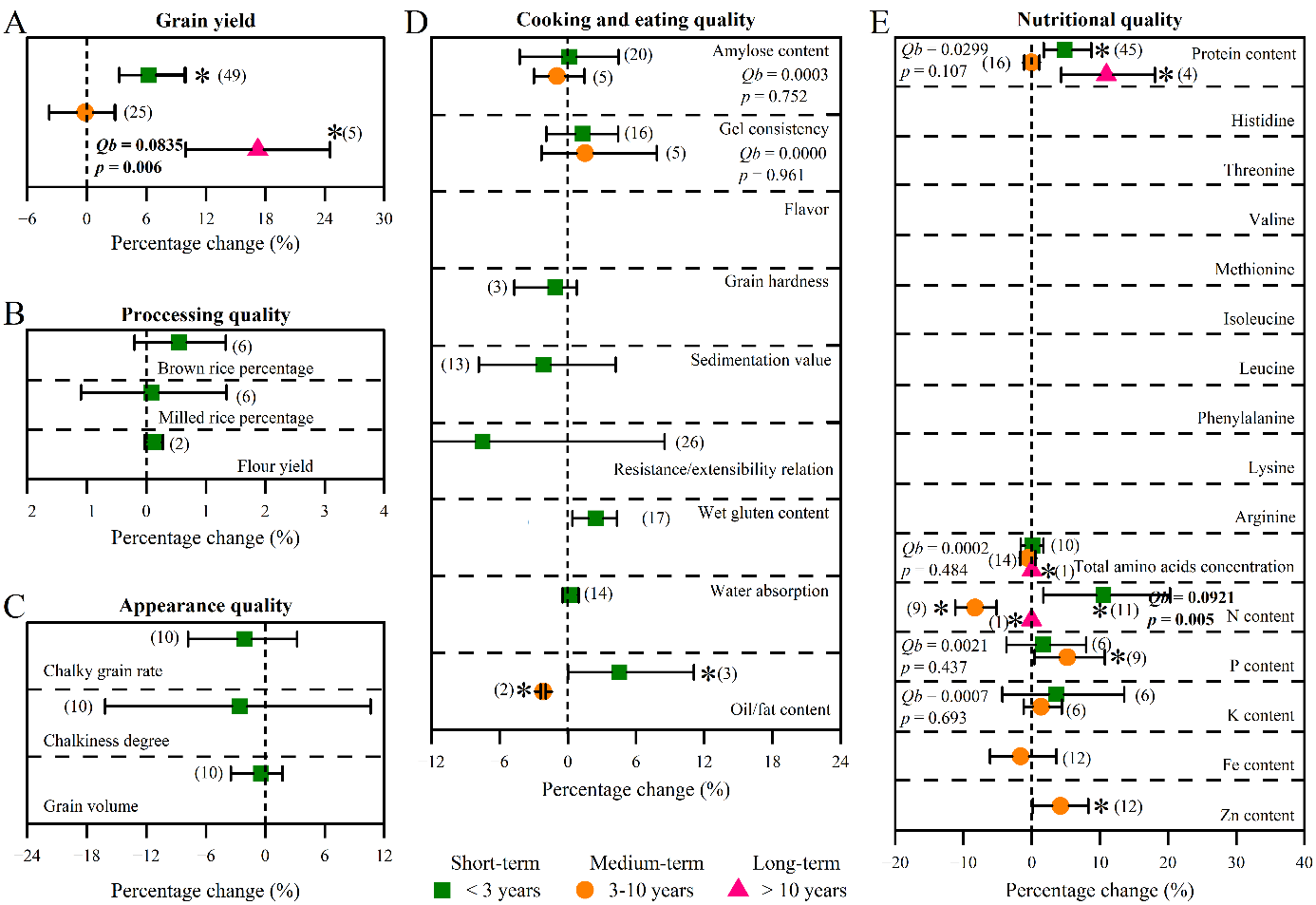


**Fig. S3** Effects of straw return on grain yield (A), processing quality (B), appearance quality (C), cooking and eating quality (D), and nutritional quality (E) under different durations of straw return (< 3 years, 3-10 years, and > 10 years). Between-group heterogeneity (*Qb*) represents the effects of categorical variables. Significant *Qb* values (*p* < 0.05) indicate that the effects of categorical variables were significant. When the 95%*CIs* did not overlap with zero, the responses of variables to overall fertilization were considered as statistically significant (*p* < 0.05, with asterisks). The numbers in brackets represent the number of observations.


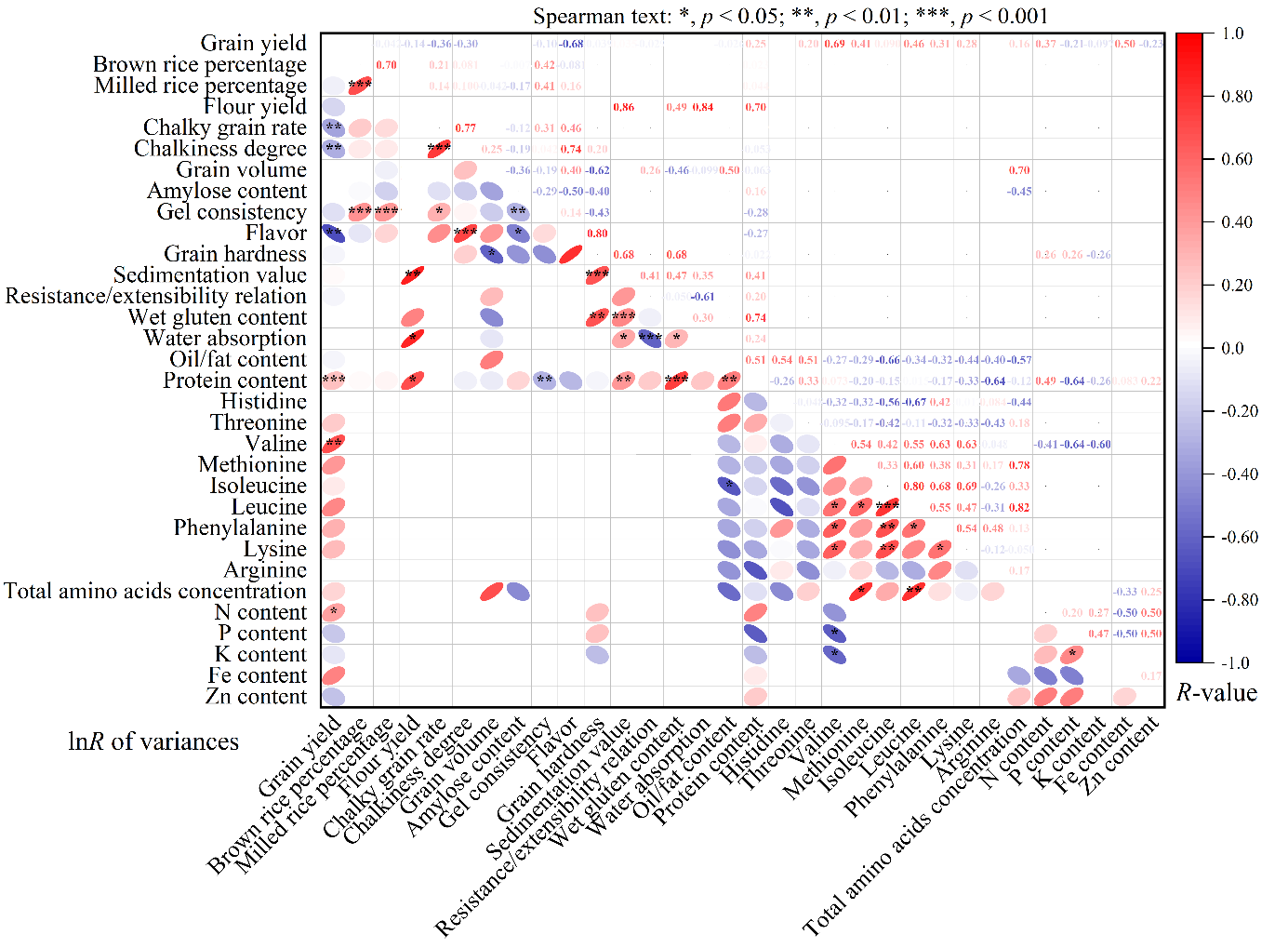


**Fig. S4** The linear relationship among the effect size of variances with the Spearman test. A positive (negative) *R*-value denotes a positive (negative) relationship (*, *p* < 0.05; **, *p* < 0.01; ***, *p* < 0.001).

**Table S1** Comparison between previous studies on the effects of straw return on crop grain yield and this study.

| **Author** | **Treatment vs. Control** | **Overall** | | **Maize** | | **Rice** | | **Wheat** | |
| --- | --- | --- | --- | --- | --- | --- | --- | --- | --- |
|  |  | *N* | Change | *N* | Change | *N* | Change | *N* | Change |
| Ding et al. (2018) | Straw return vs. Straw removal |  |  |  |  | 288 | 5.4% |  |  |
| Han et al. (2018) | Straw incorporation vs. Straw removal | 75 | 13.4% |  |  |  |  |  |  |
| Zhao et al. (2019) | Straw incorporation vs. Straw removal | 601 | 12.3% |  |  |  |  |  |  |
| Liu et al. (2019) | Crop straw retention to field vs. Straw no retention | 2092 | 8.1% | 515 | 7.2% | 342 | 7.6% | 368 | 4.1% |
| Qi et al. (2019) | Crop residue returning vs. No crop residue returning |  |  |  |  |  |  | 351 | 8.3% |
| Qin et al. (2021) | Straw mulching vs. No straw returning |  |  | 197 | 9.0% |  |  |  |  |
| Qin et al. (2021) | Straw incorporation vs. No straw returning |  |  | 118 | 10.5% |  |  |  |  |
| Wang et al. (2021) | Straw mulching vs. No straw returning |  |  | 44 | 7.3% |  |  | 52 | 3.5%^ns^ |
| Fan et al. (2021) | Straw return vs. Straw removal | 45 | 3.3% |  |  |  |  |  |  |
| Huang et al. (2021) | Straw mulching vs. No straw returning | 152 | 10.5% |  |  |  |  |  |  |
| Islam et al. (2022) | Straw return vs. Straw removal in mono-cropping systems | 256 | 11.8% | 218 | 12.5% |  |  | 38 | 8.0% |
| Islam et al. (2022) | Straw return vs. Straw removal in double-cropping systems | 815 | 5.5% | 336 | 7.9% |  |  | 479 | 3.7% |
| Qin et al. (2022) | Straw mulching vs. No straw returning |  |  |  |  |  |  | 376 | 30.0%^ns^ |
| Han et al. (2022) | Straw mulching vs. No straw returning |  |  |  |  |  |  | 97 | 14% |
| Zhang et al. (2022) | Straw return vs. No straw return |  |  |  |  | 1801 | 18.0% |  |  |
| Yao et al. (2022) | Additional crop residues vs. No additional crop residues | 17 | 3% |  |  |  |  |  |  |
| Liu et al. (2023a) | Straw return vs. No straw return | 1245 | 7.7% | 608 | 8.7% | 236 | 5.0% | 401 | 8.1% |
| Liu et al. (2023b) | Crop residue return vs. No crop residue return | 390 | 6.9% |  |  | 54 | 5.6% |  |  |
| Zhang et al. (2023a) | Straw return vs. No straw return | 72 | 9.0% |  |  |  |  |  |  |
| Zhang et al. (2023b) | Straw return vs. No straw return | 30 | 10.0% |  |  |  |  |  |  |
| Li et al. (2023) | Straw amendment vs. No-straw amendment | 1002 | 8.9% |  |  | 70 | 6.4% |  |  |
| Liu et al. (2024) | Straw incorporation vs. Inorganic fertilizer application | 207 | 7.7% |  |  | 76 | 5.1% |  |  |
| **This study** | **Straw return vs. No straw return** | **280** | **4.3%** | **39** | **6.1%** | **123** | **6.9%** | **118** | **1.2%^ns^** |

Note: *N* means the number of paired observations. “ns” means the effect of straw return was not significant (*p* > 0.05).

**Reference:**

Ding W, Xu X, He P, Ullah S, Zhang J, Cui Z, et al. Improving yield and nitrogen use efficiency through alternative fertilization options for rice in China: A meta-analysis. *Field Crops Research*, 2018, 227, 11-18.

Fan X, Chen Z, Niu Z, Zeng R, Ou J, Liu X, et al. Replacing synthetic nitrogen fertilizer with different types of organic materials improves grain yield in China: A meta-analysis. *Agronomy-Basel*, 2021, 11, 2429.

Han X, Feng Y, Zhao J, Ren A, Lin W, Sun M, et al. Hydrothermal conditions impact yield, yield gap and water use efficiency of dryland wheat under different mulching practice in the Loess Plateau. *Agricultural Water Management*, 2022, 264, 107422.

Han X, Xu C, Dungait JAJ, Bol R, Wang X, Wu W, et al. Straw incorporation increases crop yield and soil organic carbon sequestration but varies under different natural conditions and farming practices in China: A system analysis. *Biogeosciences*, 2018, 15, 1933-1946.

Huang T, Yang N, Lu C, Qin X, Siddique KHM. Soil organic carbon, total nitrogen, available nutrients, and yield under different straw returning methods. *Soil & Tillage Research*, 2021, 214, 105171.

Islam MU, Guo Z, Jiang F, Peng X. Does straw return increase crop yield in the wheat-maize cropping system in China? A meta-analysis. *Field Crops Research*, 2022, 279, 108447.

Li P, Zhang A, Huang S, Han J, Jin X, Shen X, et al. Optimizing management practices under straw regimes for global sustainable agricultural production. *Agronomy-Basel*, 2023, 13, 710.

Liu D, Song C, Xin Z, Fang C, Liu Z, Xu Y. Agricultural management strategies for balancing yield increase, carbon sequestration, and emission reduction after straw return for three major grain crops in China: A meta-analysis. *Journal of Environmental Management*, 2023a, 340, 117965.

Liu J, Fang L, Qiu T, Chen J, Wang H, Liu M, et al. Crop residue return achieves environmental mitigation and enhances grain yield: A global meta-analysis. *Agronomy for Sustainable Development*, 2023b, 43, 78.

Liu P, He J, Li H, Wang Q, Lu C, Zheng K, et al. Effect of straw retention on crop yield, soil properties, water use efficiency and greenhouse gas emission in China: A meta-analysis. *International Journal of Plant Production*, 2019, 13, 347-367.

Liu R, Hu Y, Zhan X, Zhong J, Zhao P, Feng H, et al. The response of crop yield, carbon sequestration, and global warming potential to straw and biochar applications: A meta-analysis. *Science of the Total Environment*, 2024, 907, 167884.

Qi G, Kang Y, Yin M, Ma Y, Bai Y, Wang J. Yield responses of wheat to crop residue returning in China: A meta-analysis. *Crop Science*, 2019, 59, 2185-2200.

Qin X, Huang T, Lu C, Dang P, Zhang M, Guan X-k, et al. Benefits and limitations of straw mulching and incorporation on maize yield, water use efficiency, and nitrogen use efficiency. *Agricultural Water Management*, 2021, 256, 107128.

Qin Y, Chai Y, Li R, Li Y, Ma J, Cheng H, et al. Evaluation of straw and plastic film mulching on wheat production: A meta-analysis in Loess Plateau of China. *Field Crops Research*, 2022, 275, 108333.

Wang H, Zheng J, Fan J, Zhang F, Huang C. Grain yield and greenhouse gas emissions from maize and wheat fields under plastic film and straw mulching: A meta-analysis. *Field Crops Research*, 2021, 270, 108210.

Yao C, Wu X, Bai H, Gu J. Nitrous oxide emission and grain yield in Chinese winter wheat-summer maize rotation: A meta-analysis. *Agronomy-Basel*, 2022, 12, 3168.

Zhang L, Wang Y, Lou Z, Hsu L, Chen D, Piao R, et al. Meta-analysis of factors affecting c-n fractions and yield of paddy soils by total straw return and N fertilizer application. *Agronomy-Basel*, 2022, 12, 2305.

Zhang S, Zhu Q, de Vries W, Ros GH, Chen X, Muneer MA, et al. Effects of soil amendments on soil acidity and crop yields in acidic soils: A world-wide meta-analysis. *Journal of Environmental Management*, 2023a, 345, 118531.

Zhang Y, Wang W, Li S, Zhu K, Hua X, Harrison MT, et al. Integrated management approaches enabling sustainable rice production under alternate wetting and drying irrigation. *Agricultural Water Management*, 2023b, 281, 108265.

Zhao B, Wang X, Ata-Ul-Karim ST, Liu Z, Duan A. Effect of straw incorporation on corn yield in north China: A meta-analysis. *Journal of Biobased Materials and Bioenergy*, 2019, 13, 532-536.

**Text S1** List of 78 publications on grain yield and quality as affected by straw return from which data were extracted for this analysis.

Almaz. M. G., Halim R. A., Yusoff M. M., et al. 2017. Effect of incorporation of crop residue and inorganic fertilizer on yield and grain quality of maize. Indian Journal of Agricultural Research, 51 (6), 574-579.

Arshadullah M., Ali. A., Hyder. S. I., et al. 2012. Effect of wheat residue incorporation along with n starter dose on rice yield and soil health under saline sodic soil. Journal of Animal and Plant Sciences, 22 (3), 753-757.

Babulicova, M. 2008. Influence of fertilization on winter wheat in crop rotations and in long-term monoculture. Plant Soil and Environment, 54 (5), 190-196.

Cao Y. Y., Zhang L., Ge C. B., et al. 2016. Effect of different straw and fertilization methods on ginger wheat production in lime concretion black soil (in Chinese). Tianjin Agricultural Sciences, 22 (5), 21-26.

Chen M., Li X. F., Cheng J. Q., et al. 2017. Effects of total straw returning and nitrogen application regime on grain yield and quality in mechanical transplanting japonica rice with good taste quality (in Chinese). Acta Agronomica Sinica, 43 (12), 1802-1816.

Chen P. F., Gu J. R., Han L. Y., et al. 2014. Effects of returning wheat straw to farmland and irrigation pattern on grain setting traits and quality of super rice (in Chinese). Chinese Journal of Eco-agriculture, 22 (5), 543-550.

Chen T. Z., Li B., Feng X., et al. 2016. Effects of deep buried straw and plastic film mulch on the soil moisture, yield and quality of corn (in Chinese). Journal of Shenyang Agricultural University, 47 (4), 493-498.

Fang J. J., Ding W. T., Wu X. P., et al. 2020. Effects of long-term straw and fertilizer combined application on soil nutrient, wheat yield and quality (in Chinese). Soil and Fertilizer Sciences in China, 5, 141-146.

Ge, L. L., Ma Y. H., Bian J. L., et al. 2013. Effects of returning maize straw to field and site-specific nitrogen management on grain yield and quality in rice (in Chinese). Chinese Journal of Rice Science, 27 (2), 153-160.

Gu K. J., Zhang S. M., Gu D. X., et al. 2015. Effects of rice straw returning and compaction on grain yield and quality of wheat (in Chinese). Journal of Nuclear Agricultural Sciences, 29 (11), 2192-2197.

Gupta R. K., Kaur J., Kang J. S., et al. 2022. Tillage in combination with rice straw retention in a rice-wheat system improves the productivity and quality of wheat grain through improving the soil physio-chemical properties. Land, 11 (10), 1693-1693.

Hamoud Y. A., Shaghaleh H., Ruke W., et al. 2022. Wheat straw burial improves physiological traits, yield and grain quality of rice by regulating antioxidant system and nitrogen assimilation enzymes under alternate wetting and drying irrigation. Rice Science, 29 (5), 473-488.

He G., Wang Z. H., Li F. C., et al. 2016. Nitrogen, phosphorus and potassium requirement and their physiological efficiency for winter wheat affected by soil surface managements in dryland (in Chinese). Scientia Agricultura Sinica, 49 (9), 1657-1671.

He H. X., Wang Z. H., Bao M., et al. 2018. Effects of cultivation patterns on wheat yield and grain nutrient concentration in dryland (in Chinese). Chinese Journal of Applied Ecology, 29 (3), 818-826.

He H., Huang S., Cai S., et al. 2015. Effects of nitrogen management on grain yield and quality of late rice under full amount of straw return (in Chinese). Acta Agriculturae Universitatis Jiangxiensis, 37 (3), 385-391.

Hou P. F., Ding Y. F., Zhang G. F., et al. 2015. Effects of rice or wheat residue retention on the quality of milled japonica rice in a rice–wheat rotation system in China. The Crop Journal, 3 (1), 67-73.

Hua L., Wang X. H., Zhang F. D., et al. 2008. Effect of long-term combined application of different fertilizer on yield and quality of spring wheat (in Chinese). Xinjiang Agricultural Sciences, 45 (4), 695-699.

Huang M., Wu J. Z., Li Y. J., et al. 2018. Effects of tillage method and straw mulching on grain yield and protein content in wheat and soil nitrate residue under a winter wheat and summer soybean crop rotation in drylands (in Chinese). Acta Prataculturae Sinica, 27 (9), 34-44.

Huang T., Xu L. B., Wang C. H., et al. 2015. The effects of rapid decomposition and returning of straw to the field for three consecutive years on rice yield, soil fertility, and rice quality (in Chinese). Shanghai Agricultural Science and Technology, 6, 117-118.

Qu H. F., Zhao H.B., Liu J. F., et al. 2017. NPK requirements and their physiological efficiencies for winter wheat under different cover measures in dryland (in Chinese). Journal of Plant Nutrition and Fertilizer, 23 (4), 874-882.

Jiang X. D., Chi S. Y., Ning T. Y., et al. 2010. Effects of straw returning and nitrogen applied amount on yield and quality of wheat and maize (in Chinese). Journal of Henan Agricultural Sciences, 12, 44-47.

Jiang X., Chi S. Y., Li Z. J., et al. 2007. Effects of different tillage patterns on grain quality of winter wheat (in Chinese). Transactions of the Chinese Society of Agricultural Engineering, 23 (7), 54-57.

Kang, S., Pan G. Y., Li Y. Q., et al. 2014. Effect of different fertilizer treatments on yield and quality of wheat (in Chinese). Journal of Anhui Agricultural University, 41 (4), 540-544.

Kaur J., Mahal S. S., Kaur A. 2017. Grain quality and nutrient uptake evaluation of wheat (Triticum aestivum) under different sowing methods, mulch levels and irrigation schedules. Indian Journal of Agricultural Biochemistry, 30 (1), 56-61.

Khan Ismail., Luan C., Qi W., et al. 2023. The residual impact of straw mulch and biochar amendments on grain quality and amino acid contents of rainfed maize crop. Journal of Plant Nutrition, 46 (7), 1283-1295.

Li L., Li Q. K., Lin Z. H., et al. 2021. Effects of nitrogen deep placement coupled with straw incorporation on grain quality and root traits from paddy fields. Crop Science, 61 (5), 3675-3686.

Li S. Z., Fan T. L., Zhao G., et al. 2018. Effects of different cultivation patterns on soil moisture, temperature, yield and quality of dryland maize (in Chinese). Acta Prataculturae Sinica, 27 (4), 34-44.

Li. H. Y. 2020. The influence of different fertilization modes on maize yield and quality (in Chinese). Bulletin of Agricultural Science and Technology, 8, 83-85.

Ling, Y., Zhang X. Yang J. et al. 2013. Effects of different cultivation methods and straw incorporation on grain yield and nutrition quality of rice (in Chinese). Acta Agronomica Sinica, 39 (2), 350-359.

Liu A. K., Shi D. L., Wang X. L., et al., Effects of returning straw and biochar to the field on the mineralization of paddy soil organic carbon and rice yield and quality (in Chinese). Journal of Mountain Agriculture and Biology, 40 (4), 38-45.

Liu H. Y., Hua L., Han X., et al. 2013. Effect of different methods of N application on inter-annual yield of maize, plant N uptake in loamy meadow soils (in Chinese). Journal of Shenyang Agricultural University, 44 (4), 475-478.

Liu S. P., Nie X. T., Dai Q. C., et al. 2007. Effects of interplanting with zero tillage and wheat straw manuring on rice growth and grain quality (in Chinese). Chinese Journal of Rice Science, 21 (1), 71-76.

Lv Y. J., Wang Y. J., Wang L. C., et al. 2019. Straw return with reduced nitrogen fertilizer maintained maize high yield in Northeast China. Agronomy, 9 (5), 229.

Meng X. Q., Wang Y. D., Cao G. J., et al. 2020. Effects of zinc application under straw returning on yield and zinc absorption and distribution of spring maize (in Chinese). Journal of Northwest A & F University, 48 (3), 82-90.

Qiao Y. Q., Cao C. Y., Zhao Z., et al. 2013. Effects of straw-returning and n-fertilizer application on yield,quality and occurrence of fusarium head blight of wheat (in Chinese). Journal of Triticeae Crops, 33 (4), 727-731.

Qu H., Zhao B. Q., Chen Y., et al. 2004. Effect of long-term fertilization on wheat quality and yield in grey desert soil (in Chinese). Plant Nutrition and Fertitizer Science, 10 (1), 12-17.

Randhawa. J., Sharma. R., Gurbax. S., et al. 2020. Effect of integrated nutrient management on productivity and quality of malt barley (Hordeum distichon L.). Agricultural Science Digest, 40 (3), 265-269.

Shao Y., Li J. Y., Ma G. Q., et al. 2022. The effects of long-term positioning based application of inorganic and organic fertilizers on soil nutrients and wheat grain yield and quality (in Chinese). Journal of Henan normal university, 50 (3), 126-134.

Tajnek A., Nikolic E. P., Ceh B., et al. 2010. Baking quality of wheat grain with regard to agrotechnical arrangements and location. Archives of Agronomy and Soil Science, 56 (5), 589-603,

Tan D. 2019. Winter wheat grain yield and quality response to straw mulching and planting pattern. Agricultural Research, 8 (4), 548-552.

Tang Y. L. Li C. S., Wu C., et al. 2012. Long-lasting effect of year-round tillage patterns on yield and grain quality of wheat in chengdu plain of china (in Chinese). Scientia Agricultura Sinica, 45 (18), 3721-3732.

Tang Y. L., Wu X. L., Li C. S., et al. 2013. Long-term effect of year-round tillage patterns on yield and grain quality of wheat. Plant Production Science, 16 (4), 365-373.

Tian J. Y., Xing Z. P., Li S. P., et al. 2022. Influence of wheat straw return on yield and grain quality in different direct-seeding rice production systems. Agronomy-basel, 12 (12), 3180.

Wang C. X., Song C. Y., Zhu K. L., et al. 2022. Effects of corn straw returning plus nitrogen fertilizer on starch properties and processing quality of wheat (in Chinese). Shandong Agricultural Sciences, 54 (2), 104-109.

Wang F. Y., Chen G. J., Sun L. M., et al. 2022. Interaction between tillage modes and nitrogen application rates, effects on the growth, yield and quality of wheat (in Chinese). Chinese Agricultural Science Bulletin, 38 (9), 20-26.

Wang F., Wang L. M., He C. M., et al. 2022. Effects of continuous return of Milk Vetch (Astragalus sinicus L.) and organic materials on stable yield, improved quality and efficiency in yellow-mud paddy field (in Chinese). Soils, 54 (3), 455-463.

Wang J., Lin Q., Ni Y. J., et al. 2009. Effects of different conservation tillage patterns on grain quality of winter wheat (in Chinese). Journal of Triticeae Crops, 29 (5), 881-884.

Wang S. Y., Wu J. F., Huang S., et al. 2018. The effects of total rice straw incorporation on rice growth characteristics and rice quality (in Chinese). Acta Agriculturae Universitatis Jiangxiensis, 40 (3), 454-463.

Wang T. C., Wei L., Tian Y., et al. 2009. Influence on yield and quality on the farmland scale of winter wheat-summer maize double cropping system (in Chinese). Journal of Maize Sciences, 17 (5), 108-112.

Wang Y. H., Li J. C., Wei F. Z., et al. 2013. Effects of continuous straw return with fertilizer on maize grain filling characteristics, yield and grain quality (in Chinese). Journal of Anhui Agricultural University, 40 (6), 1022-1027.

Wang, J., Qiu Y. Y., Zhang X. Y., et al. 2023. Increasing basal nitrogen fertilizer rate improves grain yield, quality and 2-acetyl-1-pyrroline in rice under wheat straw returning. Frontiers in Plant Science, 13, 1099751.

Wu Y. H., Wang L., Cui Y. Z., et al. 2021. Rice yield,quality,and soil fertility in response to straw incorporation and rotation pattern (in Chinese). Journal of Plant Nutrition and Fertitizer, 27 (11), 1926-1937.

Xin L., Liu J. T., Liu S. T., et al. 2016. Effects of combined application of straw and organic fertilizer on grain yield and quality under wheat maize rotation system (in Chinese). Acta Agriculturae Boreali-Sinica, 31 (6), 164-170.

Xu G. W., Wu C. F., Liu H., et al. 2006. Effect of straw residue returned and site-specific nitrogen management on yield and quality of rice (in Chinese). Chinese Agricultural Science Bulletin, 10, 209-215.

Xu Z. X. 2009. Effects of different fertilizer treatments on yield and quality of rice-wheat cropping (in Chinese). Acta Agriculturae Shanghai, 25 (3), 82-84.

Yan F. J., Sun Y. J., Ma J., et al. 2015. Effects of wheat straw mulching and nitrogen management on grain yield, rice quality and nitrogen utilization in hybrid rice under different soil fertility conditions (in Chinese). Chinese Journal of Rice Science, 29 (1), 56-64.

Yan F. J., Sun Y. J., Xu H., et al. 2018. Effects of wheat straw mulch application and nitrogen management on rice root growth, dry matter accumulation and rice quality in soils of different fertility. Paddy and Water Environment, 16 (3), 507-518.

Yang H. M., Wang T., Dou Y. X., et al. 2022. Effect of different mulching methods on nitrogen utilization of winter wheat in dryland (in Chinese). Journal of Triticeae Crops, 42 (8), 1012-1018.

Yuan L., Zhang Z. C., Cao X. C., et al. 2014. Responses of rice production, milled rice quality and soil properties to various nitrogen inputs and rice straw incorporation under continuous plastic film mulching cultivation. Field Crops Research, 155, 164-171.

Yuan W., Chen W. H., Wang Z. Y., et al. 2021. Effects of returning double-season rice straw to the field and reducing N-fertilizer on yield and quality of rice (in Chinese). Acta Agriculturae Universitatis Jiangxiensis, 43 (4), 711-720.

Zamir M. S. I., H.M.R. Javeed., W. Ahmed., et al. 2013. Effect of tillage and organic mulches on growth, yield and quality of autumn planted maize (Zea Mays L.) and soil physical properties. Cercetari Agronomice in Moldova, 46 (2), 17-26.

Zeng Y. H., Wu J. F. Pan X. H., et al. 2013. Study on yield and quality of double cropping rice in different straw return approaches (in Chinese). Plant Nutrition and Fertitizer Science, 19 (3), 534-542.

Zeng Y. Y., Wu J. F., He H., et al. 2011. Effect of mechanized total returning of straw to field on growth, yield and quality of early rice (in Chinese). Acta Agriculturae Universitatis Jiangxiensis, 33 (5), 840-844.

Zhang C. H., Yang S. J., Gu K. J., et al. 2013. Effect of straw-returning on carbon and nitrogen assimilate translocation and yeild formation in wheat (in Chinese). Acta Agriculturae Boreali-sinica, 28 (6), 214-219.

Zhang F., Li P., Zhang F. Y., et al. 2011. Effects of corn stalk returning on yield and quality of different types of wheat (in Chinese). Shandong Agricultural Sciences, 3, 30-32+36.

Zhang K., Zou B., Song H., et al. 2019. The effect of farming methods and straw returning on yield and quality of wheat in north of Anhui Province (in Chinese). Journal of Anhui Agricultural University, 46 (1), 111-116.

Zhang L. J., Lu Q. L., Wang H. X., et al. 2019. Effect of mulching modes on soil water and temperature, grain quality and yield of different dryland winter wheat (in Chinese). Acat Agriculturae Boreali-occidentalis Sinica, 28 (6), 888-897.

Zhang L. J., Zhang Y. H., Lu Q. L., et al. 2017. Effect of tillage model and nitrogen rate on grain quality of dryland winter wheat (in Chinese). Journal of Nuclear Agricultural Sciences, 31 (8), 1567-1575.

Zhang X. L., Zhang X., Wang Q., et al. 2010. Effects of straw returned plus nitrogen ferilizer on summer maize yield and grain quality (in Chinese). Journal of Henan Agricultural Sciences, 9, 69-73.

Zhang Y. L., Lu J. L., Jin J. Y., et al. 2012. Effects of chemical fertilizer and straw return on soil fertility and spring wheat quality (in Chinese). Plant Nutrition and Fertitizer Science, 18 (2), 307-314.

Zhang Z. C., Duan H., Yang L., et al. 2008. Effect of non-flooded cultivation patterns for the seedling-raising and transplanting of rice on grain quality and its relations with the concentrations of hormones in grains (in Chinese). Scientia Agricultura Sinica, 41 (5), 1297-1307.

Zhang Z. C., Li H. W., Wang X. M., et al. 2011. Effect of non-flooded straw-mulching cultivation on grain yield and quality of direct-seeding rice (in Chinese). Acta Agronomica Sinica, 37 (10), 1809-1818.

Zhang Z. C., Wang Z. Q., Liu L. J., et al. 2015. Effect of direct-seeding with non-flooding and wheat residue returning patterns on grain yield and grain quality of rice (in Chinese). Acta Agriculturae Boreali-Sinica, 30 (4), 168-173.

Zhang Z. C., Zhang S. F., Yang J. C., et al. 2008. Yield, grain quality and water use efficiency of rice under non-flooded mulching cultivation. Field Crops Research, 108 (1), 71-81.

Zhao P., Fu. C., 2008. Short-term influences of straw and nitrogen cooperation on nitrogen use and soil nitrate content in North Henan (in Chinese). Journal of China Agricultural University, 13 (4), 19-23.

Zhao Z. W., Li M. Y., Wu Q., et al. 2022. Effects of different soil moisture-holding strategies on growth characteristics, yield and quality of winter-seeded spring wheat. Argonomy-Basel, 12 (11), 2746.

Zhou D. D., Zhang J., Li F. J., et al. 2021. Effect of straw returning and seed dressing with chemicals on grain yield and quality of wheat following rice (in Chinese). Journal of Triticeae Crops, 41 (11), 1392-1402.

Zhou D., Zhang J., Li F. J., et al. 2022. Effects of rice straw-returning and tillage modes on yield formation and grain quality of winter wheat (in Chinese). Journal of Triticeae Crops, 42 (10), 1273-1282.
